# Ocoxin Oral Solution Triggers DNA Damage and Cell Death in Ovarian Cancer

**DOI:** 10.3390/nu16152416

**Published:** 2024-07-25

**Authors:** Sheila Almaraz-Postigo, Eduardo Sanz, Atanasio Pandiella, Elena Díaz-Rodríguez

**Affiliations:** 1 Instituto de Biología Molecular y Celular del Cáncer, Consejo Superior de Investigaciones Científicas (CSIC), Instituto de Investigación Biomédica de Salamanca (IBSAL) and Centro de Investigación Biomédica en Red Cáncer (CIBERONC), Campus Miguel de Unamuno, 37007 Salamanca, Spain; salmaraz@usal.es (S.A.-P.); atanasio@usal.es (A.P.); 2 Catalysis S.L., 28016 Madrid, Spain; eduardo@catalysis.es; 3 Department of Biochemistry, Universidad de Salamanca, 37008 Salamanca, Spain

**Keywords:** ovarian cancer, OOS, antioxidants, DNA damage, cell death

## Abstract

Ovarian cancer is the most fatal of all the reproductive cancers within the female population, mainly due to its late diagnosis that limits surgery and medical treatment. Classically, ovarian cancer therapy has included conventional chemotherapy, and other therapeutic approaches are now being used to treat these patients, but the outcomes of the disease are still poor. Therefore, new strategies are needed to improve life expectancy and life quality of ovarian cancer patients. Considering that, we investigated the effect of the nutritional supplement Ocoxin Oral Solution (OOS) in ovarian cancer models. OOS contains several nutritional supplements, some of them with demonstrated antitumoral action. *In vitro* studies showed that OOS inhibited the proliferation of several ovarian cancer cell lines, especially of those representative of the endometrioid subtype, in a time- and dose-dependent manner. A fast cell death induction after OOS treatment was observed, and when the molecular mechanisms leading to this effect were investigated, an activation of the DNA damage checkpoint was detected, as shown by activation (phosphorylation) of CHK1 and CHK2 kinases that was followed by the phosphorylation of the target protein histone H2AX. When tested in animal models of ovarian cancer, OOS reduced tumor growth without any observed secondary effects. Moreover, such reduction in tumor proliferation was caused by the induction of DNA damage as corroborated by the *in vivo* phosphorylation of CHK2 and Histone H2AX. Finally, OOS potentiated the action of carboplatin or olaparib, the standard of care treatments used in ovarian clinics, opening the possibility of including OOS in combination with those standard of care agents in patients with ovarian cancer.

## 1. Introduction

Worldwide, ovarian cancer is diagnosed every year in 295,000 women, and 185,000 of them will die from this malignancy [1,2], being that it is the most fatal of all reproductive cancers and the fifth most common cause of cancer-associated death in woman. This poor outcome is due to the lack of effective screening procedures combined with nonspecific symptoms of the disease that, altogether, cause the diagnosis of the disease to occur at advanced stages, limiting possibilities for surgery and medical treatment [2,3].

Classically, ovarian cancer therapy has included surgical resection and chemotherapy regimens including platinum or taxanes [4]. Recently, alterations in key genes involved in DNA repair have been found in ovarian cancer, opening the possibility of using inhibitors of the poly-ADP ribose polymerase (PARP), such as Olaparib or rucaparib, for the therapy of this disease [5,6,7]. Similarly, agents targeting angiogenesis such as bevacizumab have also shown efficacy in this context [8]. Within the most recent incorporations to the ovarian cancer clinic are antibody-drug conjugates, such as mirvetuximab-soravtansine. This drug targets the folate receptor alpha through the monoclonal antibody mirvetuximab. Upon internalization and processing, the antibody-drug conjugate releases the antimicrotubular agent soravtansine into ovarian cancer cells [9,10]. Besides, immunotherapeutic agents such as ipilimumab or nivolumab are also under investigation in this pathology, both alone and in combination [11,12]. Even though all these different strategies have successfully been incorporated to the clinic [13], ovarian cancer still represents a non-curable disease, mainly when diagnosed in advanced stages, making necessary the search for new therapeutic approaches.

Traditional medicine has been used over the centuries to treat malignances and improve general health. This has been the case with natural products such as vitamins or antioxidants, as, for example, the use of green tea extract and their flavonoids to prevent several malignances. Thus, the polyphenol quercetin, a lipophilic natural compound found, among other things, in green tea, vegetables, or fruits has demonstrated antitumoral capabilities on ovarian cancer [14]. Similarly, another component of green tea, epigallocathechin-3-gallate (EGCG) may have benefits in the prevention and treatment of several tumors [15,16], including reproductive system cancers [17,18].

Ocoxin Oral Solution (OOS) is a nutritional supplement composed of several plant extracts and natural products that have demonstrated antitumoral action on different models [19], including acute myeloid leukemia, hepatocellular carcinoma, glioblastoma, breast, lung, colorectal or pancreatic cancer [20,21,22,23,24]. Its composition includes plant extracts from licorice, cinnamon or green tea, several amino acids and vitamins B and C, among others [19]. Mechanistically, OOS action involves an increase in cell death together with a decrease in cell proliferation modulated by the retinoblastoma pathway and the up-regulation of p27 [19,25]. Additionally, an action as a stimulator of the immune system preventing viral infections has also been reported for OOS [26]. Based on these results, the antitumoral action of OOS was tested *in vitro* on several ovarian cancer models. OOS had a remarkable antitumoral effect due to rapid induction of DNA damage responses and cell death.

## 2. Materials and Methods

### 2.1. Reagents and Antibodies

Cell culture media (Dulbecco’s modified Eagle medium [DMEM] and Roswell Park Memorial Institute [RPMI 1640]), fetal bovine serum (FBS), penicillin and streptomycin were purchased from Invitrogen (Gaithersburg, MD, USA). Immobilon^®^-P PVDF membrane was from Merck Millipore Corp. (Darmstadt, Germany). 3-(4,5-dimethylthiazol-2-yl)-2,5-diphenyltetrazolium bromide (MTT) was from Sigma-Aldrich (St. Louis, MO, USA). Olaparib was from Selleck Chemicals GmbH (Cologne, Germany). Carboplatin was purchased from a local pharmacy. OOS was provided by Catalysis, S.L. (Madrid, Spain). Other general reagents were purchased from Millipore Corp, Sigma-Aldrich, USB Corporation (Cleveland, OH, USA), Roche Biochemicals (Hofmann, Germany), or Merck (Darmstadt, Germany).

The antibodies against BAX, MCL-1, IAP-1, AIF, PARP, CHK2, actin, calnexin and GAPDH were purchased from Santa Cruz Biotechnology (Santa Cruz, CA, USA); the anti-caspase-7, caspase-9, BAD, BID, BIM, phosphorylated Histone H2AX, phosphorylated CHK1, CHK1 and phosphorylated CHK2 from Cell Signaling Technologies (Beverly, MA, USA); the anti-caspase-3, BCL-X and XIAP from BD Biosciences (San Jose, CA, USA). The secondary HRP-conjugated antibodies against mouse or rabbit IgG were obtained from GE Healthcare Life Sciences (Piscataway, NJ, USA) and Bio-Rad Laboratories (Hercules, CA, USA), respectively. The anti-rabbit Cy3 was purchased from Jackson Immunoresearch Laboratories Inc. (West Grove, PA, USA).

### 2.2. Cell Culture

Four ovarian cancer cell lines (A2780, IGROV1, SKOV3 and OVCAR8) were cultured in DMEM or RPMI with high glucose (4500 mg/L) and antibiotics (100 mU/mL penicillin, 100 μg/mL streptomycin) and complemented with 10% FBS. Cell lines obtained from the ATCC (Manassas, VA, USA) were grown at 37 °C in a humidified atmosphere in the presence of 5% CO_2_ and 95% air.

Where indicated, cells at 70% confluence were treated with different concentrations of OOS depending on the experiment for the indicated times and collected for further analyses.

### 2.3. Protein Extraction and Western Blotting

Protein extraction and Western Blot assays were carried out as described [20]. ChemiDoc Detection System (Hercules, CA, USA) was used for protein band visualization. Densitometric measurements of the bands were performed using the Image LabTM Software Version 6.0.1 Bio-Rad Laboratories (Hercules, CA, USA). Actin, GAPDH and calnexin were used as loading controls.

### 2.4. Cell Proliferation, Cell Cycle, and Apoptosis Analyses

Proliferation analyses were performed by MTT or cell counting assays. For the latter, cells seeded in 6-well plates were treated with a complete medium containing OOS at different concentrations (1:25, 1:50, 1:100, 1:250, 1:500, 1:1000 and 1:2000) 24 h after plating, and counted in a Z1 Coulter Particle Counter (Beckman Coulter, Pasadena, CA, USA). For MTT assays, cells were plated in 24-well plates and treated as previously indicated. Cell proliferation was analyzed at 1, 2 and 3 days, when media was replaced with 250 μL of fresh medium containing MTT (0.5 μg/μL) and incubated at 37 °C for 1 h. MTT-formazan crystals were dissolved in DMSO for 10 min, and absorbance was measured at 570 nm in a plate reader (Tecan ULTRA Evolution, Männedorf, Switzerland).

For cell cycle and apoptosis analysis, cells cultured in 60 mm plates were treated with 1:100 OOS for the indicated times. For cell cycle analysis, cells were fixed in ice-cold 70% ethanol overnight. Cells were then resuspended in PBS with 500 μg/mL DNAse-free RNAse and propidium iodide (PI, 5 μg/mL) for 2 h at 37 °C. DNA content was determined in an Accuri C6 Flow Cytometer (BD).

For apoptosis assays, the protocol of the FITC-Annexin V Apoptosis Detection Kit I (BD Biosciences) [21] was followed and samples were acquired in an Accuri C6 Flow Cytometer (BD). In both experiments, 50,000 cell events were analyzed.

### 2.5. Immunofluorescence Microscopy

Ovarian cancer cell lines were cultured on glass coverslips and treated with 1:100 OOS for the indicated times. Cells were washed with PBS supplemented with 1 mM CaCl_2_, 0.5 mM MgCl_2_ and fixed using 2% paraformaldehyde. Coverslips were quenched (50 mM NH_4_Cl) and permeabilized (0.1% triton, 0.2% BSA) before blocking non-specific binding sites (PBS/CM + 0.2% BSA) for 1 h. Next, primary antibodies (pH2AX 1:200 or pCHK2 1:200) were added for 2 h at room temperature and an anti-rabbit Cy3 secondary antibody was used. Nuclei were counterstained with DAPI before mounting and confocal microscopy (Leica TCS SP5 system, Leica Microsystems CMS, Wetzlar, Germany) was used to visualize the samples.

### 2.6. In Vivo Experiments

All animal experiments were carried out according to the institutional guidelines and protocol approved by the Ethics Committee of Universidad de Salamanca (Salamanca, Spain, approval code: CBE-2019-325). For the *in vivo* experiments, 12 6-week-old female athymic mice BALB/C nu/nu (18–20 g, Charles River Laboratories, Wilmington, MA, USA) were maintained at the Universidad de Salamanca Animal Care Facility (Salamanca, Spain) in pathogen-free housing. After 1 week of quarantine, mice were injected with 3 × 10^6^ A2780 cells resuspended in DMEM and Matrigel mixture (1:1) into both caudal flanks. Tumors, once engrafted, were randomly assigned into two groups (six per group), which received either vehicle alone (PBS, control group), or 100 µL OOS per 20 g weight. Treatments were administered daily by oral gavage until the human endpoint was achieved (tumor volume, 2.000 mm^3^). Animals were sacrificed by isofluorane overdose. Tumors were measured with a digital caliper (Proinsa, Vitoria, Spain) three times per week and tumor volumes were calculated using the following formula: V = (L/2) × (W/2)2 × 4/3 × π, where V = volume (mm^3^), L = length (mm) and W = width (mm). Animal weight was similarly measured three times per week. After mice sacrifice, tumors were resected and frozen at −80 °C or fixed for further analyses.

### 2.7. Histological and Immunohistochemical (IHC) Analyses

IHC assays were performed as described [21]. The antibodies used for these histological analyses were an anti-Ki67 (Master Diagnóstica, Granada, Spain) (1:50 dilution) and pH2AX (1:100 dilution). Results were evaluated by independent pathologists from the pathology unit of the University of Salamanca (Salamanca, Spain). Positive cells were counted for each preparation in 10 different fields at 40× magnification, and three independent samples of each condition were analyzed. These analyses were performed using the Image J 1.44p Software.

### 2.8. Statistical Analyses

In proliferation experiments, each condition was analyzed in triplicate and data presented as mean ± SD of a representative experiment repeated at least 3 times. Western Blot experiments were repeated at least 3 independent times. Statistical analyses were carried out using GraphPad Prism 8 (GraphPad Software, Inc., San Diego, CA, USA). Both in the proliferation experiments and in the Western Blot, the comparison between groups were carried out using ANOVA analyses. Comparison of continuous variables between two groups in the *in vivo* experiments were performed using Student’s *t*-test. Differences were considered statistically significant when the *p*-value was less than 0.05.

## 3. Results

### 3.1. Action of OOS on Ovarian Cancer Cells

**Figure 1:**
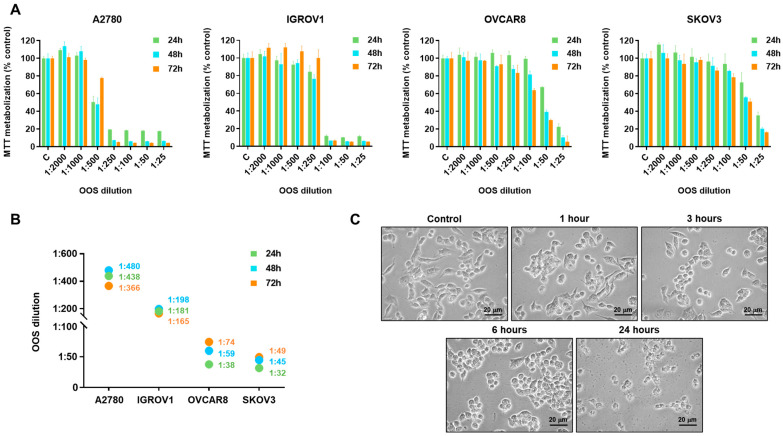
Efficacy of OOS on several ovarian cancer cellular models. (**A**) Dose and time-dependent effect of OOS on the proliferation of A2780, IGROV1, OVCAR8 and SKOV3 cells was assessed *in vitro*. Cells were incubated with OOS at the indicated dilutions, and MTT metabolization was measured at 24 (green bars), 48 (blue bars) and 72 (orange bars) hours. Mean absorbance values of untreated samples were considered as 100%, and mean values for the different treatments referred to that. Data are represented as mean ± SD of triplicates of an experiment that was repeated at least twice. (**B**) IC_50_ values of OOS (as dilution factor) for the different cell lines and times analyzed, calculated from data shown in A. (**C**) Microscopical analysis of OOS effect on A2780 cells. Cells were plated and, once attached, treated with a 1:100 dilution of OOS in culture media. Phase contrast images of the cultures were acquired at the indicated times. Scale bar: 20 μm.

To explore the potential antitumoral action of OOS in ovarian cancer, the *in vitro* effect of OOS on the proliferation of ovarian cancer cells was explored. To that end, dose-response studies were performed using four human ovarian cancer cell lines: the ovarian endometrioid carcinoma cell lines A2780 and IGROV1, the high-grade serous carcinoma cell line OVCAR8 and the ovarian serous cystadenocarcinoma cell line SKOV3. OOS was added to culture media at various dilutions and its effect on the proliferation of the ovarian cancer cell lines were analyzed by MTT metabolization studies. OOS dose-dependently inhibited the proliferation of the four ovarian cancer cell lines (Figure 1A). The antiproliferative effect of OOS was more pronounced in the endometrioid carcinoma cell lines A2780 and IGROV1 than in the high grade serous (OVCAR8) and the cystadenocarcinoma (SKOV3) cell lines. A2780 resulted as the most sensitive cell line as indicated by the IC_50_ values (Figure 1B). In that cell line, a 1:250 dilution of OOS provoked the maximal inhibitory effect on cell proliferation. Morphologically, OOS caused rounding of the A2780 cells and, after 24 h of treatment, evident signs of cell death, suggested by the presence of cell detritus and lack of refringence of the cells (Figure 1C).

**Figure 2:**
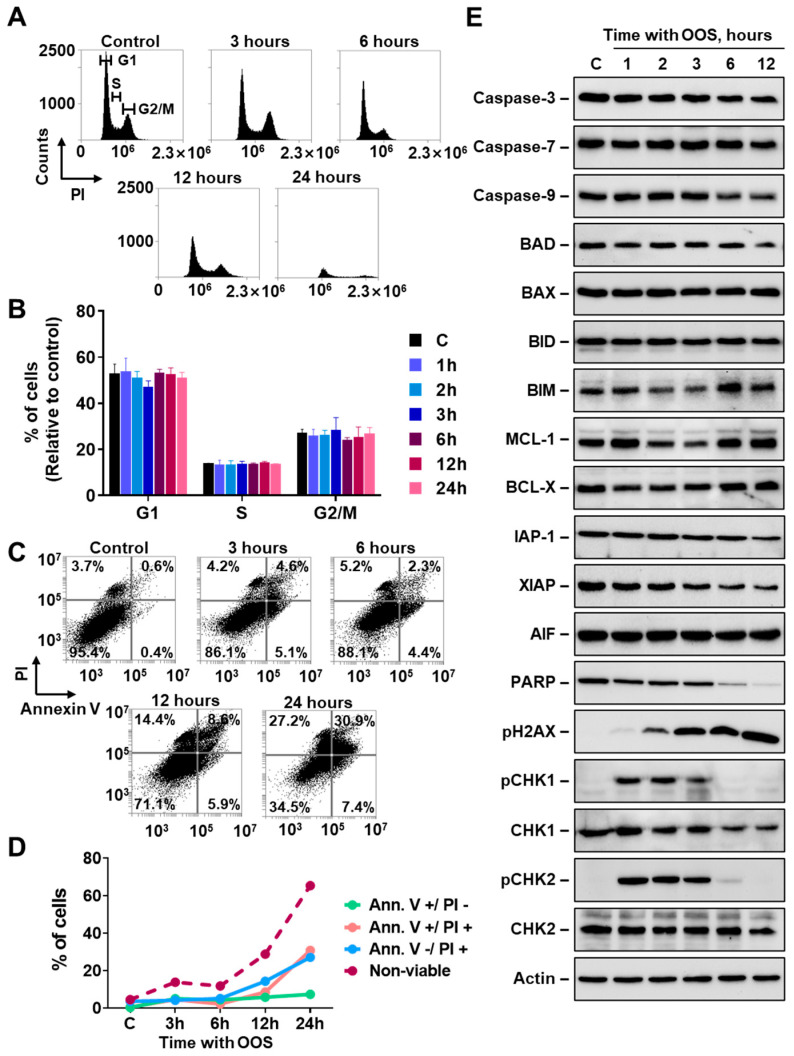
Molecular characterization of OOS effect on A2780 cells. (**A**) OOS action on cell cycle profiles in A2780 cells. A2780 cells were incubated in the presence of OOS (1:100 dilution) for the indicated times, harvested and fixed as described. Cell cycle profile after PI staining was analyzed by flow cytometry. For each time point, the percentage of cells in the different stages of the cell cycle was determined and represented in (**B**), where mean values ± SD of three independent experiments are plotted. (**C**) OOS causes rapid cell death of A2780 cells. Cells were prepared and treated as in A, were harvested, and cell death was analyzed by double staining with Annexin V-FITC/PI. The percentage of cells going through the apoptotic process (Ann V+/PI−; Ann V+/PI+; Ann V−/PI+, and the three subgroups together named as non-viable) were plotted in (**D**) for each timepoint. (**E**) OOS induces activation of the DNA damage checkpoint. A2780 cells were treated with OOS (1:100 dilution) for the indicated times, protein extracts were prepared and resolved by SDS-PAGE. The levels and status of the indicated proteins were analyzed by Western blot with specific antibodies. Actin levels were determined and considered as a loading control protein.

The reduction in MTT metabolization caused by OOS in the ovarian cancer cell lines could be due to a decrease in proliferation, an increase in cell death, or both. To explore the mechanism of the antiproliferative action of OOS we selected A2780 cells, as they represented the most sensitive cell line to OOS. To analyze whether OOS exerted its antiproliferative action by affecting cell cycle progression, A2780 cells were treated with the compound, and cell cycle profiles were analyzed by PI staining. As shown in Figure 2A,B, the percentage of cells in the different cell cycle phases was not affected by the treatment with OOS. These studies suggested that OOS did not substantially affect the distribution of the cells in the different cell cycle phases. Of note, the histograms obtained at long incubation times, particularly at 24 h, showed a decrease in the cell number caused by OOS, indicative of cell death. To explore the action of OOS on cell death, cytometric analyses using Annexin V-propidium iodide double staining was performed in A2780 cells treated with OOS for different periods of time. As shown in Figure 2C,D, treatment with OOS caused a time dependent increase in the number of cells stained by either Annexin V, propidium iodide, or both. Quantitative analyses showed that OOS rapidly increased the amount of death cells, being detected as soon as after 3 h of treatment with the product (Figure 2C,D). After 24 h of treatment with OOS, the percentage of non-viable cells was above 60%.

### 3.2. OOS Activates the DNA Damage Response in Ovarian Cancer Cells

Taken together, the above data indicated that the action of OOS on the ovarian cancer cell line A2780 was mainly caused by an induction of cell death mechanisms. We then decided to explore the biochemical consequences of OOS treatment on proteins involved in apoptotic pathways. OOS provoked a time-dependent decrease in PARP, as well as a small decrease in caspase 3, caspase 9 and XIAP (Figure 2E). The levels of the Bcl family proteins analyzed did not change very much upon treatment of the cells with OOS. The strongest changes observed corresponded to the phosphorylation status of proteins involved in DNA damage response. In fact, treatment with OOS caused phosphorylation of CHK1 and CHK2 at the earliest time point analyzed, which corresponded to an incubation time of 1 h. In the case of pH2AX, OOS treatment already caused phosphorylation at 1 h of treatment, but the peak stimulation was delayed with respect to the peak of pCHK1 and pCHK2 (Figure 2E).

**Figure 3:**
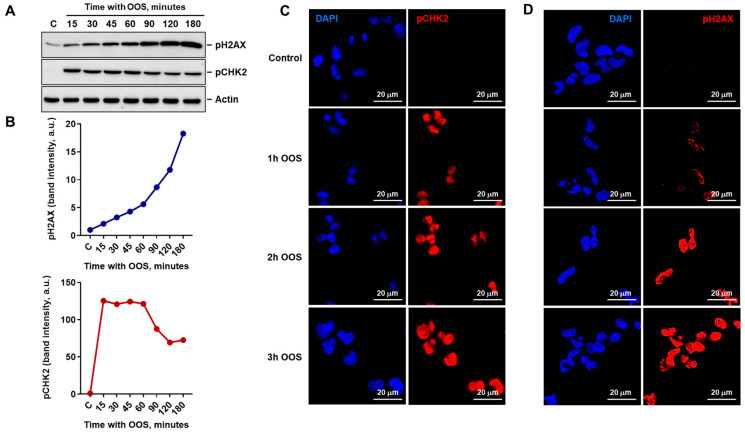
OOS activates the DNA-damage checkpoint in A2780 cells. (**A**) OOS induces rapid activation of the DNA damage checkpoint, as shown by the presence of pCHK2 and pHistone H2AX. Cells were treated for the indicated times (minutes) with OOS, protein extracts were prepared and the amount of these two markers was determined by WB with specific antibodies. Actin levels were used as loading control. (**B**) The amount of phosphorylated histone H2AX (upper graph) or CHK2 (lower graph) from (**A**) were densitometrically determined and plotted for each time point as described in the methods section; a.u.: arbitrary units. (**C**,**D**) The phosphorylation of these two markers was verified by immunofluorescence. A2780 cells were plated on glass coverslips and treated for the indicated times with OOS (1:100 dilution). pCHK2 (**C**) or pH2AX (**D**) were analyzed by IF, as described (labeled in red). Samples were counterstained with DAPI (DNA in blue). Scale bar: 20 μm.

Because of the rapid activation of pCHK1 and pCHK2 in those experiments, a shorter time course experiment which included additional intermediate times of analysis was performed. This experiment confirmed that pCHK2 was rapidly activated by OOS and anticipated the increase in pH2AX (Figure 3A,B). In fact, pCHK2 reached a peak of activation at the first time point analyzed (15 min), while in the case of pH2AX it increased progressively up to the final point analyzed (3 h) (Figure 3A,B). Immunofluorescence studies showed that staining of pCHK2 and pH2AX was restricted to the nuclear compartment and confirmed that activation of pCHK2 anticipated that of pH2AX (Figure 3C,D). Moreover, these studies showed that pH2AX distributed following a nuclear punctate pattern, more visible at early time points (Figure 3D and Figure S1).

**Figure 4:**
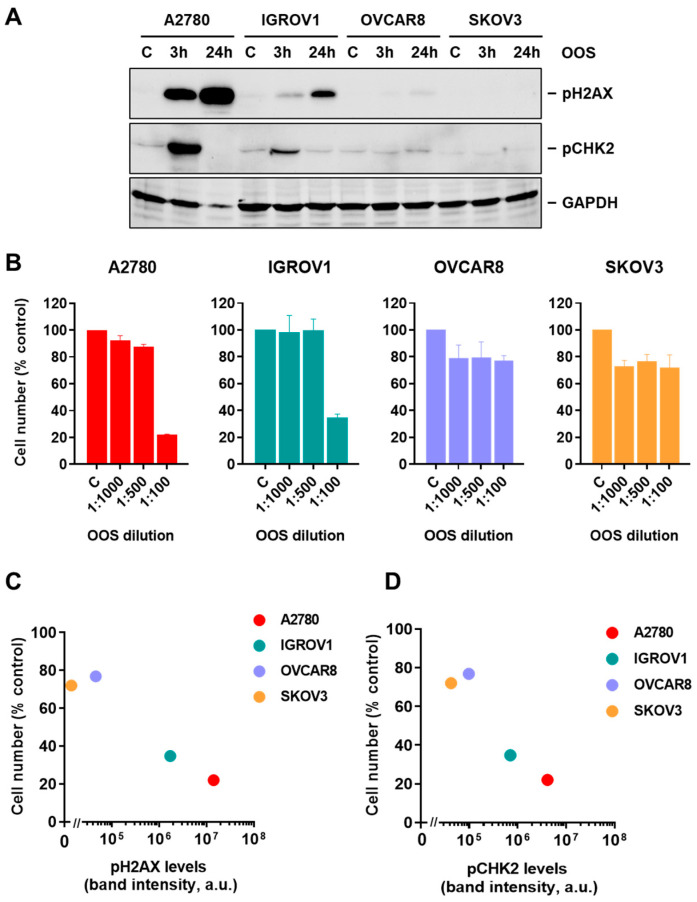
OOS action and DNA-damage checkpoint activation in ovarian cancer models. (**A**) Activation of the DNA damage checkpoint by OOS in several ovarian cancer models. The indicated ovarian cell lines were incubated for 3 or 24 h in the presence of OOS (1:100 dilution). Then, protein extracts were prepared and the amount of pH2AX or pCHK2 was determined by WB. GAPDH was used as a loading control. (**B**) Efficacy of OOS on several ovarian cancer cellular models was determined by cell counting experiments. The four ovarian cell lines were plated and grown for 24 h in the presence of the indicated dilutions of OOS, and the final number of cells was determined by direct cell counting. Mean cell numbers of untreated samples were considered to be 100%, and mean values for the different treatments referred to that. Graphs show mean ± SD of triplicates of an experiment that was repeated at least twice. (**C**,**D**) Correlation of cell numbers after OOS treatment and pH2AX (**C**) or pCHK2 (**D**). The amount of phosphorylated histone H2AX or CHK2 analyzed in A was densitometrically determined and plotted for each ovarian cell line with the corresponding cell numbers as measured in B. The graphs show the correlation between the WB values and the cell counting experiments at 24 h with the 1:100 dilution of OOS; a.u.: arbitrary units.

Next, whether activation of the DNA damage response pathway also occurred in other sensitive ovarian cancer cell lines in response to OOS was explored. To that end, the four ovarian cancer cell lines used in this study were treated with a 1:100 dilution of the compound for 3 or 24 h and the levels of pCHK2 and pH2AX were analyzed by Western blot. As shown in Figure 4A, treatment with OOS increased pH2AX and pCHK2 levels in A2780 and IGROV1. Since at that dilution the action of OOS on cell proliferation was very low (Figure 1A), the effect of OOS on the pH2AX and pCHK2 levels in OVCAR8 and SKOV3 cells was less appreciable.

To analyze whether there was a relationship between the degree of activation of the DNA damage response pathway and the antiproliferative response to OOS, the ovarian cancer cell lines were treated with several dilutions of OOS for 24 h and the effect of OOS on cell number was analyzed. In parallel, cell extracts from identically treated cultures were obtained to perform quantitative measurements of pH2AX and pCHK2 levels. The proliferation studies confirmed that A2780 and IGROV1 cells were the most sensitive cell lines with respect to the antiproliferative action of OOS (Figure 4B). Plotting of the band intensity of pH2AX versus the cell number remaining in the cultures after 24 h of treatment with a 1:100 dilution of OOS showed an inverse relationship between both parameters (Figure 4C). Similarly, representation of pCHK2 amount versus cell number showed an analogous correlation (Figure 4D).

### 3.3. OOS Augments the Antiproliferative Action of Carboplatin and Olaparib

**Figure 5:**
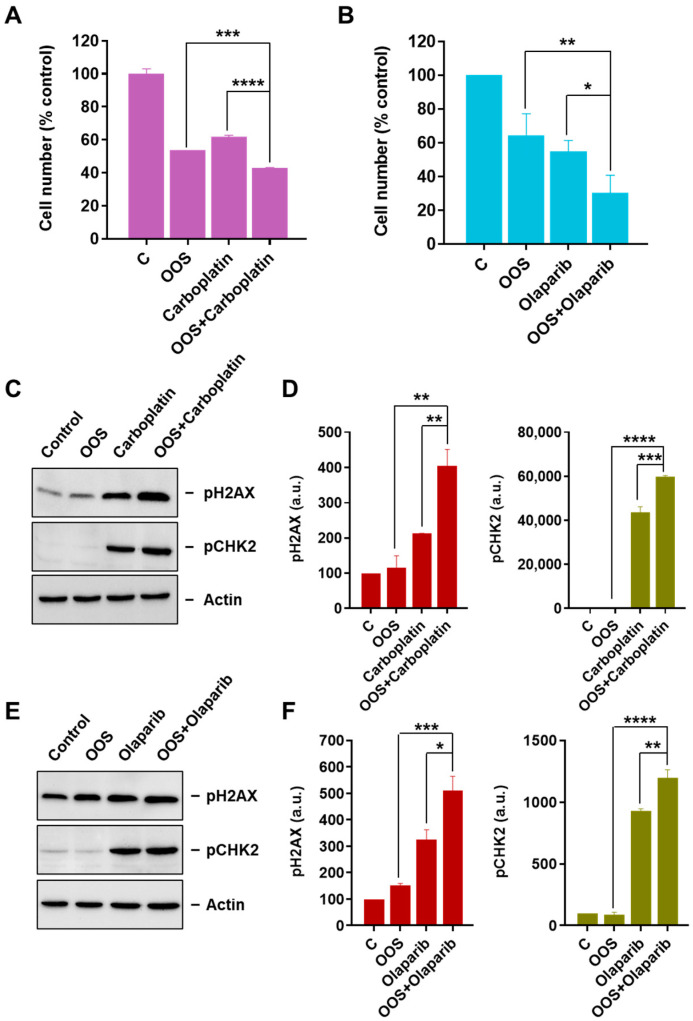
OOS potentiates the action of standard of care treatments in ovarian cancer. The effect of OOS alone (1:500 dilution) or in combination with Carboplatin (100 µM concentration, (**A**)) or Olaparib (5 µM concentration, (**B**)), were determined in cell counting experiments after 48 h of treatment, in A2780 cells. Mean cell numbers of untreated samples were considered 100%, and mean values for the different treatments referred to that. (**C**) Analysis of the DNA damage checkpoint in OOS and carboplatin combination. Cells were plated and treated as in A, and protein lysates were prepared and quantified. The amount of phosphorylated histone H2AX or CHK2 were analyzed by WB, and actin levels were determined as a loading control. Blots were scanned and pH2AX or pCHK2 were densitometrically determined and plotted (**D**). (**E**) Similarly, the combination of OOS and Olaparib was analyzed by WB and the levels of pH2AX or pCHK2 plotted (**F**). Data in this figure represent mean ± SD of an experiment that was repeated at least three times. Asterisks indicate statistical differences between the different groups, assessed with an ANOVA; a.u.: arbitrary units with respect to control values. *p*-value: <0.05 (*); <0.01 (**); <0.005 (***); <0.001 (****).

An important therapeutic strategy in oncology is the use of drug combinations to achieve better antitumoral action. To analyze whether OOS could increase the action of drugs commonly used in the therapy of ovarian cancer, combination experiments with carboplatin or Olaparib were performed. For these studies, a suboptimal dilution dose of OOS (1:500) had to be used to facilitate the analysis of a potential increase in the action of the standard of care drugs tested. In A2780 cells, OOS was able to increase the antiproliferative action of both carboplatin (Figure 5A) and Olaparib (Figure 5B). Western blot analyses showed that OOS, at the concentration used, did not substantially increase the levels of pH2AX and pCHK2 (Figure 5C–F). In contrast, carboplatin and Olaparib augmented pH2AX and pCHK2 levels, and those levels were further increased by the addition of OOS.

### 3.4. In Vivo Antitumoral Efficacy of OOS

**Figure 6:**
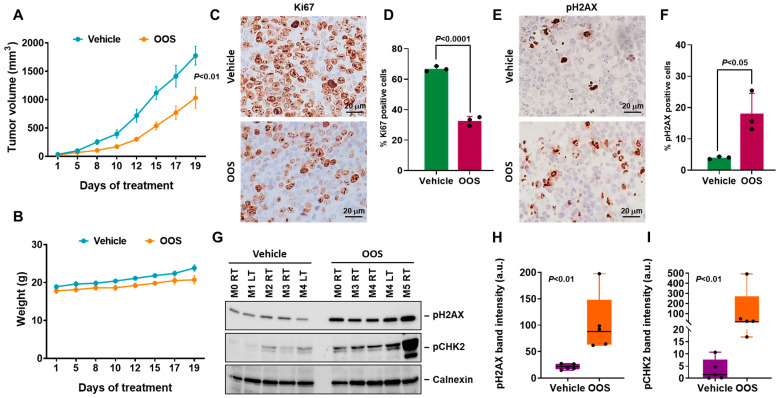
*In vivo* efficacy of OOS. (**A**) OOS interferes with tumor growth *in vivo*. Female Balb/c nu/nu athymic mice were subcutaneously injected with 3 × 10^6^ A2780 cells and, on the next day, were randomized into two groups that were treated daily by oral gavage with vehicle (blue line) or OOS (100 μL/20 g animal, orange line). Tumor volumes were measured three times per week with a caliper. Data are represented as mean tumor volume ± SEM of the tumors included in each group. (**B**) Effect of OOS treatment on animal weight was analyzed on the same days as tumor measurement. (**C**) OOS induces a reduction of cell proliferation *in vivo* as determined by Ki67 staining of the tumors. At the time of animal sacrifice, part of the different tumors was processed for IHC analysis. Ki67 marker was used to study proliferation, and 3 random tumors were stained. Pictures of representative fields of the control (upper) or OOS (lower) treated tumors are shown. Bar: 20 μm. (**D**) Quantitation of the number of Ki67 positive cells in the three independent tumors. For each tumor, the number of positive cells was assessed in 10 different arbitrary fields and plotted. Each black bullet represents the mean values of 10 fields of each sample. (**E**) OOS induces an increase of DNA damage *in vivo*. Tumor samples were processed as in C and stained for phosphorylated-Histone H2AX. (**F**) The total number of pH2AX positive nuclei were analyzed and plotted as in D. (**G**) *In vivo* activation of DNA damage checkpoint proteins by OOS. The amount of the indicated proteins was assessed in five independent tumors for each of the groups (vehicle or OOS treated) by Western. pH2AX (**H**) or pCHK2 (**I**) levels were densitometrically assessed and normalized to calnexin and plotted. Data are represented as mean ± SD of the 5 tumors analyzed and each black bullet represents one of the measures; a.u.: arbitrary units.

To explore whether the *in vitro* antiproliferative properties of OOS could be reproduced *in vivo*, A2780 cells were injected in the flanks of nude mice and randomized into two groups to be treated orally with OOS or vehicle. Individual curves for the growth of tumors in each mouse are shown in Supplementary Figure S2A and the mean values appear in Figure 6A. OOS reduced the growth rate of A2780 xenografts in a significant manner. Measurements of body weight showed that OOS-treated mice weighted similarly to the vehicle-treated animals (Figure 6B), suggesting that at the administered dose, the product did not exert major toxic effects.

Immunohistochemical analyses of A2780-derived tumors showed that treatment with OOS reduced the amount of the proliferation marker Ki67 (Figure 6C,D and Figure S2B). In addition, these studies also showed that tumors from the mice treated with OOS expressed higher levels of pH2AX than vehicle-treated controls (Figure 6E,F). Western blot analyses confirmed that OOS was able to, *in vivo*, increase the amount of pH2AX (Figure 6G,H) as well as pCHK2 (Figure 6G,I).

## 4. Discussion

Ovarian cancer still represents a difficult to treat disease, particularly when surgical treatment cannot eliminate the tumor [1]. In those advanced stages, medical therapy using a variety of drugs, including chemotherapy, biological agents or directed drugs are the remaining option [4], but they are not curative and do not prevent disease progression. Therefore, strategies to augment the current therapeutic armamentarium, or to increase its efficacy are required.

In the present study we have evaluated the antitumoral effect of OOS in ovarian cancer. The *in vitro* models used, derived from human cell lines, recapitulated three major types of ovarian cancer, namely endometrioid, high grade serous carcinoma and cystadenocarcinoma [3]. OOS induced a clear decrease in the proliferation of all the cell lines analyzed. However, it is important to mention that the effect was more pronounced in cells representative of the endometrioid subtype. Such effect on cell number was found to be both time and dose dependent. Furthermore, OOS reduced tumor progression *in vivo* in mice implanted with the endometrioid cell line A2780, which appeared to be the most sensitive cell line to OOS *in vitro*. Of note, OOS treatment slowed down tumor growth without apparent toxicity, since the weights of the control and treated mice were analogous, and no detectable changes in their behavior were observed. This was somehow expected as OOS is a nutritional supplement which has proven to be safe in preclinical models as well as in real-world experience [27,28,29,30].

OOS contains several products with demonstrated antitumoral capability (Supplementary Table S1), whose concerted action is responsible for its antitumoral properties. To gain insights into the mechanism of the antitumoral effect of OOS in ovarian cancer, several studies were carried out. To evaluate if it was due to a reduction in cell duplication, an increase in cell death or both, Annexin V staining as well as cell cycle profiling were carried out. These studies indicated that OOS triggered cell death but did not exert an appreciable effect on the cell cycle. Biochemical analyses demonstrated that OOS rapidly induced a DNA damage response, as indicated by the up regulation of pCHK1 and pCHK2 levels, followed by an increase in pH2AX, markers of that biological response [31,32,33]. The effect of OOS on pCHK2 was detected already at 15 min of treatment, suggesting that no changes in gene expression were involved in this early effect. In other disease models, such as acute myeloid leukemia [20] or hepatocellular carcinoma [21], OOS has been reported to influence cell cycle progression. In fact, OOS has been reported to increase the levels of p27^Kip1^ [25], an inhibitor of cell cycle progression, whose presence prevents the activation of the cyclin E-CDK2 and cyclin D-CDK4 complexes, and thus blocks the cell cycle at the G1 phase [34,35]. It is relevant to mention that while we were unable to detect an effect of OOS on some *in vivo* cell cycle markers, OOS was able to decrease the amount of the proliferation marker Ki67 in the tumors resected from mice, pointing to a decrease in cell proliferation in those *in vivo* conditions.

Most antitumor therapies are based on the combination of different drugs to increase the action of the individual agents [6]. Bearing this in mind, the possible potentiation of standard of care drugs used in the ovarian cancer clinic, such as carboplatin or Olaparib [36,37] by OOS was explored. When OOS was used together with these two compounds, a potentiation of their antitumoral properties was observed, opening the possibility of using OOS in patients treated with those drugs, to promote their antitumoral effect. This is especially important for patients that suffer from metastatic disease at the time of diagnosis, and in those cases where only palliative chemotherapy can be considered. For these patients, improvements of the standard of care treatment by the addition of other agents such as OOS could offer clinical benefits without increased toxicities. Obviously, a clinical trial checking this hypothesis would be valuable.

## 5. Conclusions

Ocoxin Oral Solution demonstrated antiproliferative effect on ovarian cancer cellular models. *In vitro* studies demonstrated that its action included DNA damage, as indicated by the activation of the marker p-histone H2AX. Moreover, Ocoxin Oral Solution arrested tumor cell growth *in vivo*. Finally, OOS was able to enhance the effect of antitumoral drugs used to treat ovarian cancer patients. 

## Data Availability

Data are contained within the article.

## Supplementary Materials

The following supporting information can be downloaded at: https://www.mdpi.com/article/10.3390/nu16152416/s1, Figure S1: OOS action and DNA-damage checkpoint activation by assessment of pH2AX. Histone H2AX is phosphorylated when DNA damage signaling pathway is activated after OOS treatment. Such phosphorylation occurs as fast as one hour after OOS treatment and is detected by the appearance of foci (dots) of pH2AX on the DNA. In the image, cell nuclei are stained with DAPI in blue and pH2AX in red.; Figure S2: OOS attenuates ovarian cancer tumor growth *in vivo*. (A) OOS interferes with tumor growth *in vivo*. Female Balb/c nu/nu athymic mice were subcutaneously injected with 3 × 10^6^ A2780 cells and next day randomized to two groups that were daily treated by oral gavage with vehicle (black lines) or OOS (100 μl/20 g animal, red lines). Tumor volumes were measured and plotted for each animal that is represented by an independent line. Data shown in (A) were also differentially plotted in (B) (vehicle treated controls) or (C) (OOS treated). (D) Quantification of the number of Ki67 positive cells in the three independent tumors analyzed. For each tumor, the number of positive cells was assessed in 10 different random fields and plotted. Green circles represent the untreated control tumors and purple triangles OOS treated ones. (E) Similarly, the number of pH2AX positive cells was assessed in the three independent tumors, 10 different random fields/tumor, analyzed and plotted.; Table S1: OOS composition and mechanism of action of its components. References [38,39,40,41,42,43,44,45,46,47,48,49,50,51,52,53,54,55,56,57,58,59,60,61,62,63,64] are cited in Supplementary Materials.
