# Supplementary material for: Ocoxin Oral Solution Triggers DNA Damage and Cell Death in Ovarian Cancer
Source: Nutrients. 2024 Jul 25;16(15):2416. doi: 10.3390/nu16152416 (PMC11313973; doi:10.3390/nu16152416)
Supplement: Supplementary file 1 [file nutrients-16-02416-s001.zip › nutrients-3088758-supplementary.pdf]

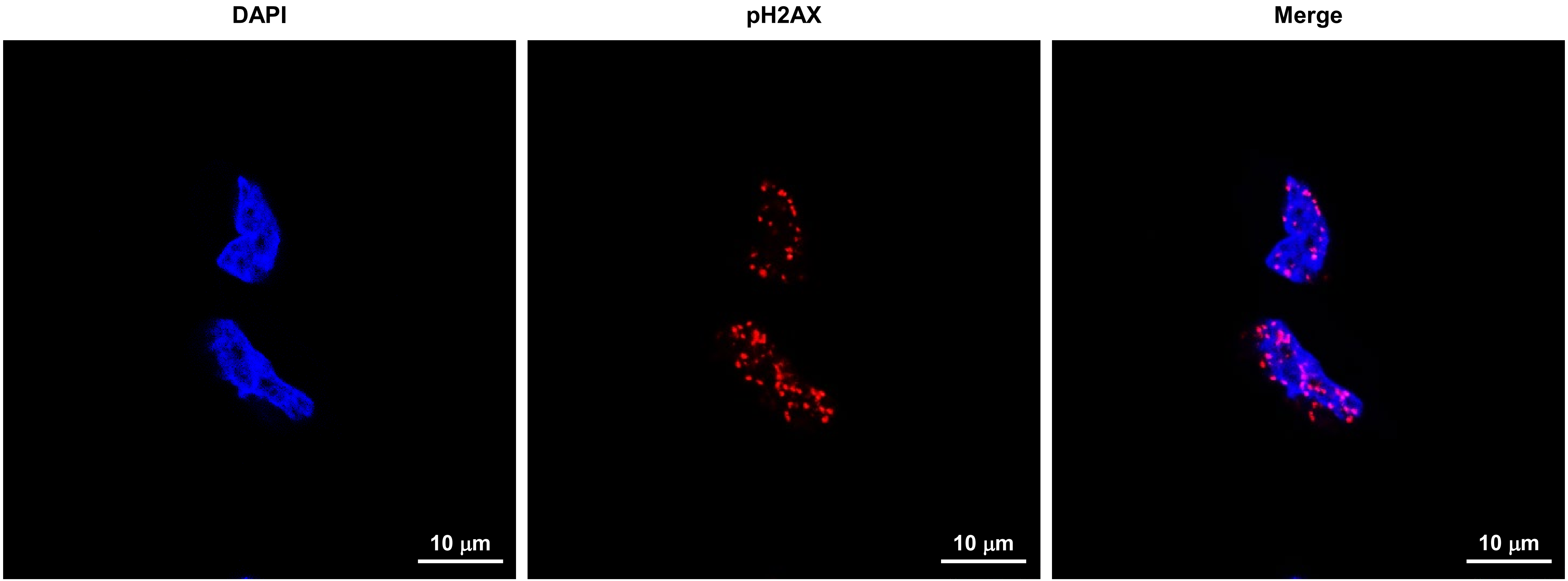

Supplementary figure S1

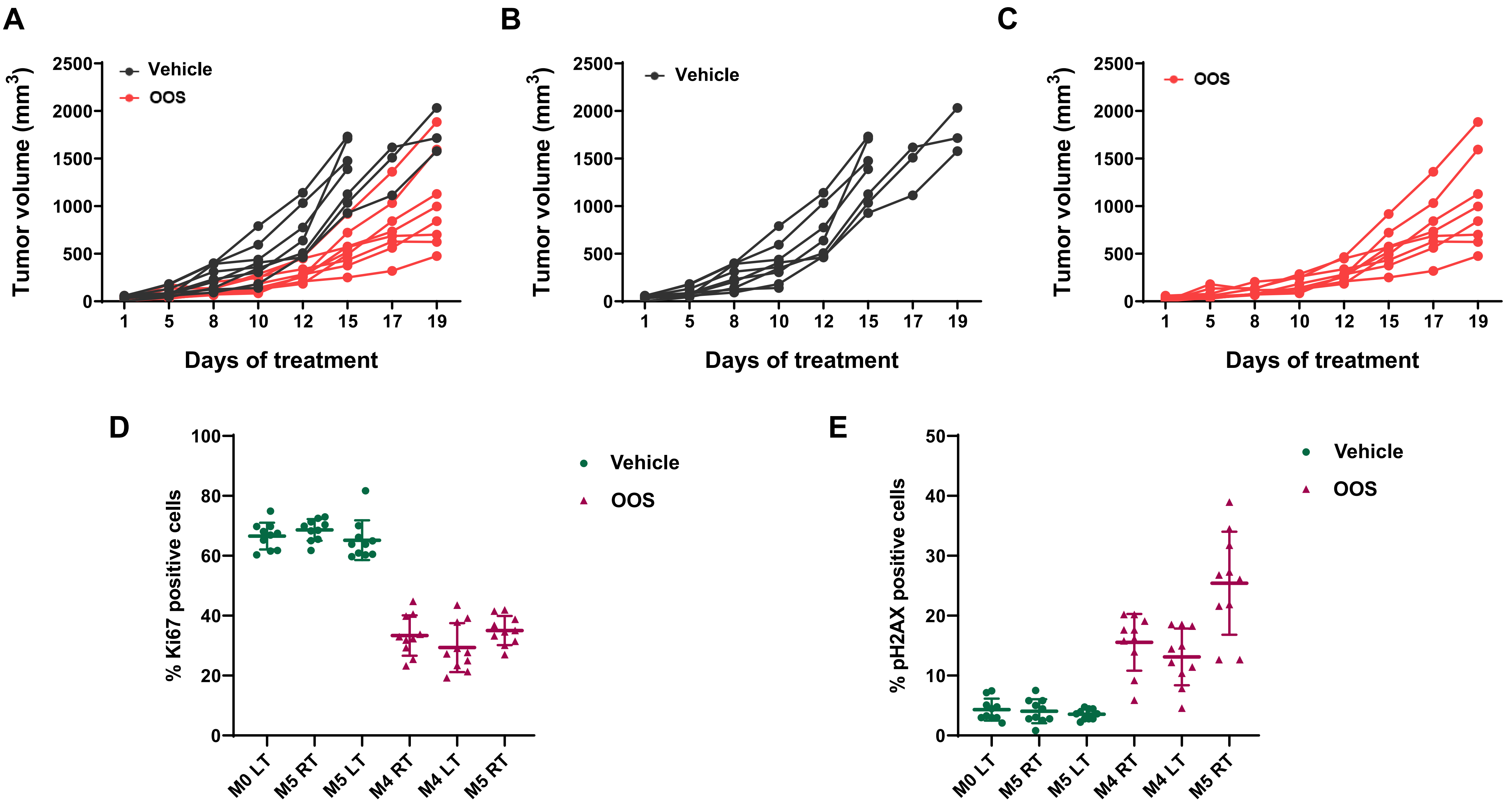

Supplementary figure S2

Supplementary Table S1. OOS composition and mechanism of action of its components

COMPOSITION	QUANTITY	ACTION AND REFERENCES
Glucosamine sulphate potassium chloride	600 mg	Reduces inflammation[38]
L-Glycine	600 mg	Cell cycle control[39,40]
L-Arginine	192 mg	Favors immune surveillance and induces apoptosis[41,42]
L-cysteine	61.2 mg	Reduces oxidative stress[43]
Glycyrrhizinic acid (Licorice Extract, *Glycyrrhiza glabra L.*)	60 mg	Cell cycle and stemness control, induces apoptosis, reduces neovascularization and metastasis[44,45,46]
L-Ascorbic Acid (Vitamin C)	36 mg	Favors immune surveillance and stemness control[47]
Sodium Benzoate	30 mg	Preservative
Potasium Sorbate	30 mg	Preservative
Zinc Sulfate	24 mg	Reduces inflammation and oxidative stress[48,49]
Passion Fruit Aroma	15 mg	Organoleptic (flavour)
Epigallocatechin-3-gallate [Green tea extract*, Camellia sinensis (L) Kuntze*]	7.5 mg	Cell cycle and stemness control, reduces neovascularization, metastasis, inflammation and oxidative stress; regulates epigenetics; and induces apoptosis[50,51,52,53,54,]
Calcium Pantothenate (Pantothenic Acid)	3.6 mg	Regulates epigenetics[55,56,57]
Manganese sulphate	1.2 mg	Induces tumoral cell apoptosis[58,59]
Pyridoxine hydrochloride (Vitamin B6)	1.2 mg	Regulates epigenetics[60]
Cinnamon extract (*Cinnamomum verum J. Presl.*)	0.9 mg	Cell cycle control and induces apoptosis[61]
Folic acid (pteroylmonoglutamic acid)	120 µg	Regulates epigenetics[62,63]
Cyanocobalamin (vitamin B12)	0.6 µg	Regulates epigenetics[64]
Malic acid	360 mg	Favors immune surveillance and reduces oxidative stress
Sucralose	7.2 mg	Reduces inflammation
Water e.q.f.	30 ml	Vehicle

38. Ma L, Rudert W, Harnaha J, Wright M, Machen J, Lakomy R et al. Immunosuppressive Effects of Glucosamine. Journal of Biological Chemistry. 2002;277(42):39343-39349.
39. Zhang Y, Jia H, Jin Y, Liu N, Chen J, Yang Y et al. Glycine Attenuates LPS-Induced Apoptosis and Inflammatory Cell Infiltration in Mouse Liver. The Journal of Nutrition. 2020;150(5):1116-1125.
40. Wang W, Wu Z, Dai Z, Yang Y, Wang J, Wu G. Glycine metabolism in animals and humans: implications for nutrition and health. Amino Acids. 2013;45(3):463-477.
41. Inoue M, Okamoto K, Terashima A, Nitta T, Muro R, Negishi-Koga T et al. Arginine methylation controls the strength of γc-family cytokine signaling in T cell maintenance. Nature Immunology. 2018;19(11):1265-1276.
42. Geiger R, Rieckmann J, Wolf T, Basso C, Feng Y, Fuhrer T et al. L-Arginine Modulates T Cell Metabolism and Enhances Survival and Anti-tumor Activity. Cell. 2016;167(3):829-842.e13.
43. Šalamon, Š., Kramar, B., Marolt, T., Poljšak, B. and Milisav, I., 2019. Medical and Dietary Uses of N-Acetylcysteine. Antioxidants, 8(5), p.111.

44. Ohtsuki and Iahida: Inhibitory effect of Glycyrrhizin on Polypeptide Phosporylation by Polypeptide-dependent Proteinase (Kinase P) in vitro. Biochem Biophys Res Commun. 1988 Dec 15;157(2):597-604.
45. Sasaki H et al: Effect of glycyrrhizin, an active component of licorice roots, on HIV replication in cultures of peripheral blood mononuclear cells from HIV-seropositive patients. Pathobiology. 2002-2003;70(4):229-36.
46. Cristina Fiore et al: Antiviral Effects of Glycyrrhiza species. Phytother. Res. 22, 141-148 (2008). Review Article.
47. Sorice A, Guerriero E, Capone F, Colonna G, Castello G, Costantini S. Ascorbic Acid: Its Role in Immune System and Chronic Inflammation Diseases. Mini-Reviews in Medicinal Chemistry. 2014;14(5):444-452.
48. Wintergerst E, Maggini S, Hornig D. Immune-Enhancing Role of Vitamin C and Zinc and Effect on Clinical Conditions. Annals of Nutrition and Metabolism. 2006;50(2):85-94.
49. Hojyo S, Fukada T. Roles of Zinc Signaling in the Immune System. Journal of Immunology Research. 2016;2016:1-21.
50. Yan YB, Tian Q, Zhang JF, Xiang Y. Antitumor effects and molecular mechanisms of action of natural products in ovarian cancer. Oncol Lett. 2020 Nov;20(5):141.
51. Ding S, Xu S, Fang J, Jiang H. The Protective Effect of Polyphenols for Colorectal Cancer. Front Immunol. 2020 Jul 10;11:1407.
52. Piwowarczyk L, Stawny M, Mlynarczyk DT, Muszalska-Kolos I, Goslinski T, Jelińska A. Role of Curcumin and (-)-Epigallocatechin-3-O-Gallate in Bladder Cancer Treatment: A Review. Cancers (Basel). 2020 Jul 5;12(7):1801.
53. Almatroodi SA, Almatroudi A, Khan AA, Alhumaydhi FA, Alsahli MA, Rahmani AH. Potential Therapeutic Targets of Epigallocatechin Gallate (EGCG), the Most Abundant Catechin in Green Tea, and Its Role in the Therapy of Various Types of Cancer. Molecules. 2020 Jul 9;25(14):3146.
54. Aggarwal V, Tuli HS, Tania M, Srivastava S, Ritzer EE, Pandey A, Aggarwal D, Barwal TS, Jain A, Kaur G, Sak K, Varol M, Bishayee A. Molecular mechanisms of action of epigallocatechin gallate in cancer: Recent trends and advancement. Semin Cancer Biol. 2020 May 24:S1044-579X(20)30107-3.
55. Hogan P. Calcium-NFAT transcriptional signalling in T cell activation and T cell exhaustion. Cell Calcium. 2017;63:66-69.
56. Nathan C. Neutrophils and immunity: challenges and opportunities. Nature Reviews Immunology. 2006;6(3):173-182.
57. Feske S. Calcium signalling in lymphocyte activation and disease. Nature Reviews Immunology. 2007;7(9):690-702.
58. HERNROTH, B., HOLM, I., GONDIKAS, A. and TASSIDIS, H., 2020. Manganese Inhibits Viability Of Prostate Cancer Cells.
59. LIU, J., GUO, W., LI, J., LI, X., GENG, J., CHEN, Q. and GAO, J., 2015. Tumor-targeting novel manganese complex induces ROS-mediated apoptotic and autophagic cancer cell death. International Journal of Molecular Medicine, 35(3), pp.607-616.
60. Li C, Huang J, Zhu H, Shi Q, Li D, Ju X. Pyridoxal-5′-Phosphate Promotes Immunomodulatory Function of Adipose-Derived Mesenchymal Stem Cells through Indoleamine 2,3-Dioxygenase-1 and TLR4/NF-κB Pathway. Stem Cells International. 2019;2019:1-15.
61. Sadeghi S, Davoodvandi A, Pourhanifeh MH, Sharifi N, ArefNezhad R, Sahebnasagh R, Moghadam SA, Sahebkar A, Mirzaei H. Anti-cancer effects of cinnamon: Insights into its apoptosis effects. Eur J Med Chem. 2019 Sep 15;178:131-140.
62. Samblas M, Martínez J, Milagro F. Folic Acid Improves the Inflammatory Response in LPS-Activated THP-1 Macrophages. Mediators of Inflammation. 2018;2018:1-8.
63. Henry C, Nemkov T, Casás-Selves M, Bilousova G, Zaberezhnyy V, Higa K et al. Folate dietary insufficiency and folic acid supplementation similarly impair metabolism and compromise haematopoiesis. Haematologica. 2017;102(12):1985-1994.
64. Calder P, Carr A, Gombart A, Eggersdorfer M. Optimal Nutritional Status for a Well-Functioning Immune System Is an Important Factor to Protect against Viral Infections. Nutrients. 2020;12(4):1181.
